# Aberrant septin 9 DNA methylation in colorectal cancer is restricted to a single CpG island

**DOI:** 10.1186/1471-2407-13-398

**Published:** 2013-08-30

**Authors:** Reinhold Wasserkort, Alexandra Kalmar, Gabor Valcz, Sandor Spisak, Manuel Krispin, Kinga Toth, Zsolt Tulassay, Andrew Z Sledziewski, Bela Molnar

**Affiliations:** 1 Epigenomics AG, Berlin, Germany; 2 Current address: Delta-Vir GmbH, Leipzig, Germany; 3 2nd Department of Internal Medicine, Semmelweis University, Budapest, Hungary; 4 Current address: Zymo Research, Irvine CA 92614, USA; 5 Epigenomics Inc, Seattle, WA 98104, USA; 6 Molecular Medicine Research Unit, Hungarian Academy of Science, Budapest, Hungary

**Keywords:** DNA methylation, Septin 9, Colorectal cancer, Adenoma, Epithelial cells, Stromal cells, Direct bisulfite sequencing, Immunohistochemistry

## Abstract

**Background:**

The septin 9 gene (SEPT9) codes for a GTP-binding protein associated with filamentous structures and cytoskeleton formation. SEPT9 plays a role in multiple cancers as either an oncogene or a tumor suppressor gene. Regulation of SEPT9 expression is complex and not well understood; however, hypermethylation of the gene was recently introduced as a biomarker for early detection of colorectal cancer (CRC) and has been linked to cancer of the breast and of the head and neck. Because the DNA methylation landscape of different regions of SEPT9 is poorly understood in cancer, we analyzed the methylation patterns of this gene in distinct cell populations from normal and diseased colon mucosa.

**Methods:**

Laser capture microdissection was performed to obtain homogeneous populations of epithelial and stromal cells from normal, adenomatous, and tumorous colon mucosa. Microdissected samples were analyzed using direct bisulfite sequencing to determine the DNA methylation status of eight regions within and near the SEPT9 gene. Septin-9 protein expression was assessed using immunohistochemistry (IHC).

**Results:**

Regions analyzed in SEPT9 were unmethylated in normal tissue except for a methylation boundary detected downstream of the largest CpG island. In adenoma and tumor tissues, epithelial cells displayed markedly increased DNA methylation levels (>80%, p <0.0001) in only one of the CpG islands investigated. SEPT9 methylation in stromal cells increased in adenomatous and tumor tissues (≤50%, p <0.0001); however, methylation did not increase in stromal cells of normal tissue close to the tumor. IHC data indicated a significant decrease (p <0.01) in Septin-9 protein levels in epithelial cells derived from adenoma and tumor tissues; Septin-9 protein levels in stromal cells were low in all tissues.

**Conclusions:**

Hypermethylation of SEPT9 in adenoma and CRC specimens is confined to one of several CpG islands of this gene. Tumor-associated aberrant methylation originates in epithelial cells; stromal cells appear to acquire hypermethylation subsequent to epithelial cells, possibly through field effects. The region in SEPT9 with disease-related hypermethylation also contains the CpGs targeted by a novel blood-based screening test (Epi *pro*Colon®), providing further support for the clinical relevance of this biomarker.

## Background

Alterations in the DNA methylation profile of cells are among the earliest molecular changes in cancer [1]. Both locus-specific hypermethylation and genome-wide hypomethylation commonly occur in different types of tumors [2]. Hypermethylation of tumor suppressor genes has been identified as a critical step in tumor initiation as the silenced expression of such genes affects whether cells maintain normal growth. Such epigenetic events, along with mutations, provide cells with a selective advantage that may lead to their clonal expansion [3].

Septin 9 (SEPT9)^a^ involvement in cancer was first discovered as a fusion product with the MLL gene in leukemia [4]. Subsequent studies showed that SEPT9 was frequently deleted in sporadic ovarian tumors [5] or amplified in breast cancer [6]; it was suggested that the gene might be a candidate ovarian tumor suppressor gene that may also act like an oncogene. A comprehensive screen of a wide variety of tissue samples and cell lines revealed that SEPT9 was ubiquitously expressed, although, its isoform expression appeared to be tissue specific [7]. Moreover, SEPT9 mRNA and protein were overexpressed in diverse human tumors further suggesting an important role of the gene in tumorigenesis.

SEPT9 belongs to a highly conserved family of septin genes coding for GTP-binding proteins. These multidomain proteins assemble into complexes and form filamentous structures which comprise part of the cytoskeleton [8,9]. The septin proteins play important roles in many cellular processes by providing rigidity to the cell membrane, serving as scaffolds to recruit proteins to specific subcellular locales, and creating membrane diffusion barriers to establish discrete cellular domains [8]. SEPT9 is characterized by its complex genomic organization that spans 219 kb and has 18 distinct transcripts encoding 15 isoforms [10,11]. Alternative splicing at both 5′ and 3′ ends generates this transcript diversity [10]. The gene also harbors several CpG islands that when methylated may impact the expression of different transcripts.

Several analyses have been conducted on the expression of the SEPT9 transcripts. The transcript SEPT9_v1 was overexpressed in breast, ovarian, and prostate cancer while no SEPT9_v1 expression was observed in the normal tissue of these organs [12,13]. Two distinct transcripts, SEPT_v4 and SEPT_v4*, code for the same polypeptide, but are translated with different efficiencies and respond differently to cellular stresses; the SEPT9_v4* polypeptide is thought to play a role in neoplasia [14,15]. The transcript SEPT9_v3 expression was up-regulated in some cancer cell lines and repressed in others in which there was also a reduction in protein levels [16]. This transcript was also associated with promoter hypermethylation.

Recently, SEPT9 was shown to be epigenetically modified in colorectal cancer (CRC) [17]. Subsequent studies using refined and more sensitive assays confirmed SEPT9 as a biomarker for CRC [18,19]. A complete workflow was developed on the basis of these findings and permits a minimally invasive, blood-based screening test (Epi *pro*Colon®) for colorectal cancer [20], which is currently available for clinical application in Europe [21]. The test detects methylated SEPT9 in circulating DNA in plasma which is believed to be derived from apoptotic cells shed from the tumor [22]. However, it is unknown whether SEPT9 hypermethylation is present in all types of tumor cells of the colon and to what extent the hypermethylation affects the promoter region of SEPT9. To advance our understanding of cancer-associated epigenetic changes in SEPT9, we investigated the methylation profile of SEPT9 in epithelial and stromal cells microdissected from normal and diseased fresh-frozen biopsies and tissue colon samples. Since earlier studies analyzed DNA methylation changes only in a single SEPT9 CpG island, and only in heterogeneous CRC tissue specimens, this study aims at determining whether the aberrant methylation changes are unique to only this CpG island or whether hypermethylation affects multiple CpG dense regions associated with this gene and how SEPT9 methylation is affected in homogeneous populations of epithelial and stromal cells.

Our results provide new insights into the cellular origin of aberrant DNA methylation in the SEPT9 gene for CRC and the clinical relevance of DNA methylation for early CRC detection.

## Methods

### Clinical data

Biopsies of normal mucosa were obtained from three patients, aged 32 to 72 during routine endoscopy examinations (Additional file 1: Table S1). Patients from whom normal tissue samples were obtained were verified to be free of bowel disease. Adenomatous polyps and tumor samples were obtained from 5 patients, aged 53 to 75 during surgical resection. Samples were obtained after informed consent and the study had been approved by the local ethics committee (TUKEB 2005/037, Semmelweis University Regional and Institutional Committee of Science and Research Ethics, Budapest, Hungary). Tissue from core tumor areas and normal tissue adjacent to tumor (NAT) from two distances to the tumor were obtained: NAT1, microscopically normal tissue from a distance of 1 cm from tumor; and NAT2, minimum of 10 cm from tumor. Surgically removed tissue samples were snap-frozen in liquid nitrogen immediately after surgery and stored at −80°C until cryosection.

### Laser capture microdissection

Laser capture microdissection (LCM) was performed on normal, adenomatous, and colorectal tumor mucosa specimens after they were embedded in optimal cutting temperature (OCT) media (Sakura, Netherlands) and prepared for cryosectioning: 6 μm sections were cut on PALM Membrane Slide 1.0 PEN (Carl Zeiss, Germany) slides at −20°C and stored at −80°C until used. Consecutive sections were used for H&E and IHC staining for LCM and photo documentation. A PALM laser microdissector (Carl Zeiss, Germany) was used to collect a total of 96 specimens with a target of approximately 1000 cells each, as shown in Additional file 2: Table S2. Dissected specimens were collected in 500 μl vials; specimens were immersed in lysis buffer containing proteinase K immediately after sectioning and kept at −80°C until further processing.

### Preanalytic processing of LCM specimens

Microdissected samples were lysed at 56°C for three hours followed by the addition of fresh proteinase K (ProtK) solution, and then overnight incubation at 37°C to ensure complete digestion of the samples. All lysates were subjected to bisulfite treatment without prior DNA extraction. To further minimize DNA loss while maintaining high DNA conversion rates a non-standard protocol was established. Bisulfite reagents (from Epi *pro*Colon® 2.0 kit, Epigenomics, Germany) were used with a modified protocol: 45 μl of bisulfite solution plus 10 μl of protection buffer were added to each lysate and incubated at 80°C for 45 minutes in a thermocycler (MJ Research Tetrad, GMI, USA). Next, binding buffer and poly-dA (Qiagen, Germany) were added to the lysate solutions, which were then placed on to silica membranes (Zymo Research, USA). On-column desulfonation using 0.2 M NaOH for two minutes was followed by two washing steps using commercial buffer solutions AW1 and AW2 (Qiagen, Germany), and finally, DNA was eluted using 40 μl of pre-warmed (50°C) PCR-grade water. Eluted DNA was stored at −20°C until used for analysis. The total amount of bisulfite-converted DNA samples was quantified using an in-house duplex qPCR assay which determines genomic and bisulfite DNA simultaneously [23]. This assay determined the median amount of DNA per specimen as 5.4 ng (Additional file 2: Table S2) corresponding to a median of around 900 cells per LCM specimen. 92% of the specimens had less than 1% detectable genomic DNA indicating a high conversion of genomic DNA to bisulfite DNA. In two LCM samples, unconverted genomic DNA comprised more than 10% of the total DNA, indicating a poor conversion. However, none of these samples were discarded as there was still a sufficient amount of converted template DNA for the bisulfite specific primers in the amplification and sequencing assays. Multiplex PCR was then performed once per sample using up to 2 ng of the respective DNA.

### Sequencing assays and methylation analysis

To best utilize the limited amounts of DNA obtained from microdissected specimens for the analysis of multiple loci, a two-step multiplex amplification protocol was adopted [23] to establish a 13-loci multiplex PCR (mPCR) (Additional file 3: Table S3). For each locus at least two primer pairs were designed and tested in singleplex PCR (sPCR) reactions. The better performing primer pair, based on agarose gel analysis, was then tested in combination with other primers. All primers were void of CpGs so that amplification was not influenced by the methylation status. PCR was performed in two consecutive steps comprising a pre-amplification (mPCR) on 2 ng bisulfite DNA from each sample, and a re-amplification step for each of the amplicons (sPCR). mPCR was done after initial denaturation for 15 minutes at 95°C for 50 cycles with the following temperature profile: 20 seconds at 95°C, 45 seconds at 58°C, and 30 seconds at 72°C. The highly stringent temperature was selected to minimize false priming in the mPCR reaction, and high cycle numbers were chosen to ensure sufficient amounts of PCR product generated under these conditions. Re-amplification of a single locus was done using the same forward primer as in the mPCR and a new reverse primer with a short sequence tag (cgtcgtcg) at the 5′end, but otherwise identical to the reverse primer of the mPCR. This tag provided each amplicon with an internal calibrator equivalent to three fully methylated CpGs. A volume of 2 μl of the mPCR product was used as the template of each sPCR, and the same cycling conditions were used for 45 cycles. All sample DNAs were subjected to one mPCR pre-amplification reaction using a single 96-well plate to minimize reaction to reaction variation. For re-amplification with sPCR, we processed all sample-specific mPCR products in parallel for each amplicon to avoid batch processing effects.

Completed PCR reactions were subjected to direct bisulfite sequencing using Sanger sequencing chemistry as previously described [24]. Capillary gel analysis of the purified products was performed on ABI 3730 XL instruments by LGC Genomics (Berlin, Germany). Resulting trace-file data were provided online and analyzed using the ESME software to display methylation status as heatmaps and numerically as previously described [25]. Numerical methylation values were obtained for each CpG and averaged from all CpGs within each amplicon. The resulting median was used to express methylation levels as a percentage value for each amplicon and sample. Since all amplicons were reamplified and sequenced only once per microdissected sample the obtained sequencing data were then averaged as shown in Additional file 2: Table S2 (number of biological replicates indicated in last column).

Artificial mixtures of bisulfite-converted methylated and unmethylated DNA were prepared to analyze the technical performance of the assays in this workflow. Bisulfite-converted fully methylated DNA from peripheral blood lymphocytes (PBLs) (Millipore, Germany) and sperm DNA, previously reported to be free of methylation at most loci [23,26], were used to prepare mixtures corresponding to 0%, 25%, 50%, 75%, and 100% methylation. Quantification of the measured methylation level was done after these known DNA standards and mixtures were processed in duplicate through all workflow steps starting from pre-amplification to sequencing. Statistical significance of methylation differences was tested using one-way ANOVA analysis based on the quantitative sequencing data obtained for each amplicon.

### IHC staining

Immunohistochemistry (IHC) staining was done as previously described [27] with modifications. Fresh-frozen sections (6 μm) were fixed in acetone for 5 minutes and air dried for 30 minutes. After fixation, the slides were washed in PBS, followed by a 10-minute incubation in 1% BSA solution to block unspecific binding. Sections were then incubated with the primary antibody against Septin-9 (polyclonal AB cat.# PAB4799, Abnova, Germany) in 1:50 dilution for 60 minutes at 37°C, washed again in PBS, and detected with Alexa Fluor 546 dye in 1:100 dilution after 30 minutes of incubating at 37°C. Staining for cell nuclei was obtained with Hoechst 33258 (Sigma Aldrich, USA) dye for 10 seconds. The slides were scanned using the Panoramic 250 FLASH scanner with the pco.edge camera at 20× magnification (PCO AG, Germany). Relative quantification of Septin-9 protein expression was done by determining Alexa Fluor 546 mean fluorescence intensity per pixel of selected area of epithelial and stromal cells (10 and 4 areas, respectively) using the Histoquant application within the Panoramic Viewer Software V1.15 (3DHISTECH Ltd., Hungary); mean fluorescence intensity per pixel was measured. Data were analyzed using one-way ANOVA.

## Results

**Figure 1:**
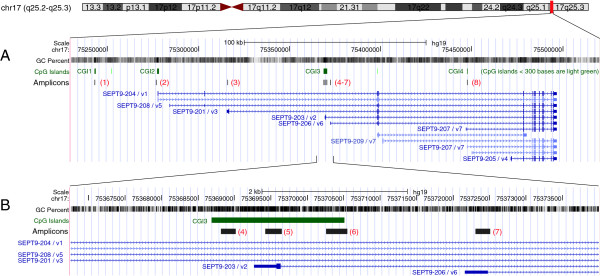
**Genomic organization of SEPT9.** The UCSC genome browser (http://genome.ucsc.edu) was used to display the location and genomic organization of SEPT9 on chromosome 17q25. **(A)** Genomic region covering approximately 300 kb: the eight amplicons (IDs given in red letters and within brackets) are shown after aligning their sequences using BLAST; CpG islands are shown in the track above the amplicons and gene transcripts are shown in the tracks below the amplicons. CGIs were numbered for easier reference within this manuscript. The UCSC display of transcripts is annotated with the combined IDs from Ensembl and NCBI/gene map for easier cross platform identification. **(B)** Detail of approximately 6 kb showing a higher resolution of the locations of the closely neighboring amplicons 4 to 7.

**Figure 2:**
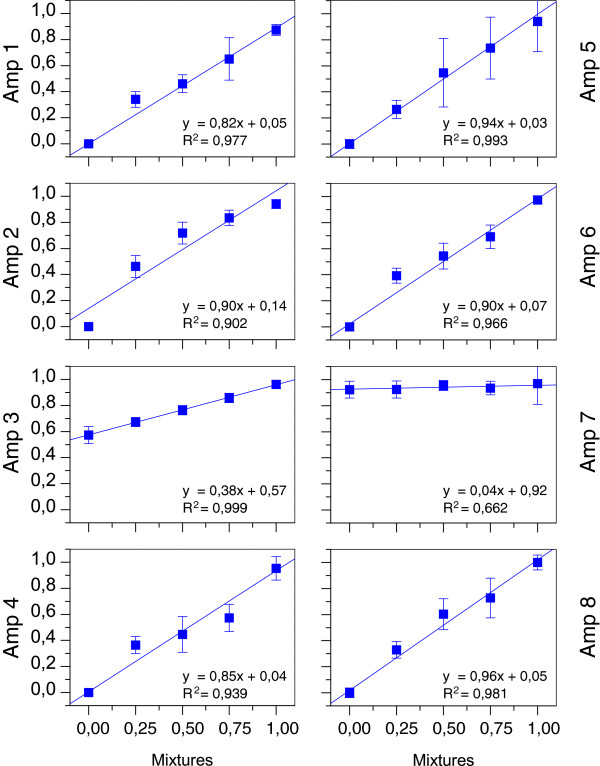
**Amplicon calibration curves.** Methylation of the artificial DNA mixtures is shown on the x-axis, and the experimentally determined methylation value for each amplicon is shown on the y-axis. Both values are given as a percentage of fully methylated DNA.

The mPCR assay established for this study included primer pairs for 13 different loci (Additional file 3: Table S3; only the results for SEPT9-related loci are presented in this paper). The eight amplicons within and near SEPT9 covered a total of 130 CpGs and were located predominantly in CpG-rich regions, except for amplicon 3, which had a lower CpG density (Figure 1 and Additional file 4: Figure S1). The suitability of the amplicons for quantitatively determining methylation levels were assessed with the calibration experiments. A slope of the linear regression curve close to one and offset values close to zero were indicative for sensitive assays. The results from these experiments showed that the methylation levels of six of the eight amplicons were well quantified while the results for amplicons 3 and 7 were ambiguous (Figure 2). Sperm DNA was considered an inappropriate control DNA for these loci because both appeared to be either fully or partially methylated. However, since the sample sequencing results obtained with these two assays were consistent and reproducible, even at lower levels of methylation, we accepted these results in the absence of complete calibration data. Together, these eight assays generated robust data from the 96 samples analyzed.

### DNA methylation of SEPT9

**Figure 3:**
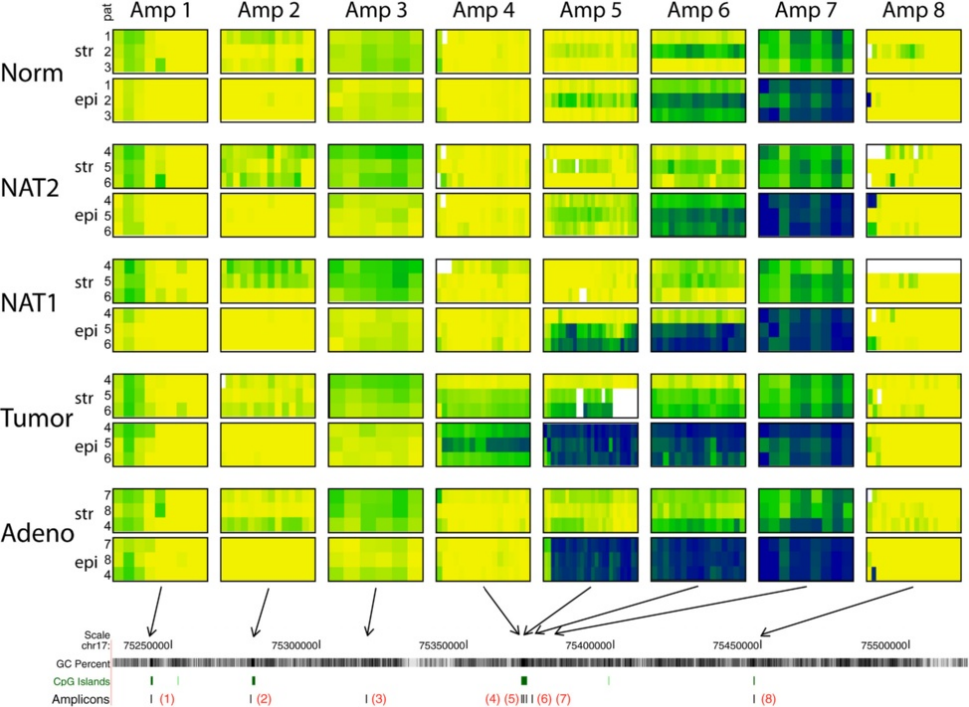
**Bisulfite DNA sequencing results for SEPT9 amplicons.** Each column displays results from one amplicon and each row displays results for one cell type. Within each of the rectangular boxes, the columns correspond to single CpGs and the three rows correspond to three patients. As all amplicons are drawn to the same scale the location of the amplicons is illustrated at the bottom with arrows pointing to the genomic map (compare to Figure 1). Color coding: yellow = unmethylated (0%), green = partially methylated (50%), blue = fully methylated (100%), and lighter or darker green/blue colors provide methylation levels below or above 50%. White areas = no sequencing data available. Abbreviations: str = stromal cells, epi = epithelial cells, Norm = normal colon tissue, NAT1 = normal adjacent tissue (1 cm away from tumor), NAT2 = normal adjacent tissue (> 10 cm from tumor), Adeno = adenoma tissue, pat = patient no.: only the last number of the patient ID in Additional file 1: Table S1 is shown here.

The results obtained from the eight amplicons within and near SEPT9 provided an overview of the methylation landscape of this gene for two different cell types (Figure 3). One extragenic and three intragenic CpG islands were designated as CGI1, CGI2, CGI3, and CGI4 (Figure 1). CGI3 was the largest of these CpG islands and previously described as differentially methylated in colorectal cancer [17]. This region was covered by three non-overlapping amplicons to obtain a higher resolution of methylation changes in the region.

**Figure 4:**
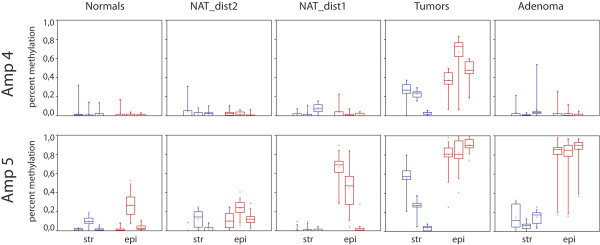
**Box plot diagrams of SEPT9 methylation.** Results from amplicons 4 and 5 in different LCM tissue specimens. Data are shown separately for stromal (str) and epithelial (epi) cells and for the three patients in each group. Red = epithelial cells, blue = stromal cells. The horizontal bar within boxes shows the median, dots within the boxes the means, the lower and upper boundaries of boxes show the 25th and 75th percentiles, the whiskers are determined by the 5th and 95th percentiles.

Disease-associated aberrant methylation was seen exclusively in CGI3 (Figure 3) while the other regions in SEPT9 did not show detectable alterations in their methylation status. Amplicon 5, located centrally in CGI3, showed large methylation differences between normal samples and those from colon adenomas and tumors; this difference was the most pronounced in epithelial cells (Figures 3 and 4). The methylation difference between normal and tumor tissue in these cells exceeded 80% and was highly significant (p <0.0001). Equally pronounced was the difference in epithelial cells between normal and adenomatous tissue. NAT1 samples (i.e., < 1 cm from the tumor) showed significantly elevated methylation levels (p <0.0001) in the epithelial cells in two of the three tumors, a finding that supports the concept of a field effect in CRC [28,29]. This effect was not seen in the third NAT1 sample. No significant methylation differences were observed between epithelial cells derived from normal and NAT2 samples (p > 0.05). In contrast, in stromal cells, an increase in methylation was seen mainly in tumor samples with changes up to 50%, while in adenomas, methylation levels increased by less than 20%. No increase was detected in stromal cells of the NAT samples. These data suggest that the observed aberrant methylation in SEPT9 - CGI3 originates in epithelial cells and is associated with progression in the adenoma-carcinoma sequence.

Our results further show spreading of aberrant methylation from the core of CGI3 towards the 5′ end which is covered by amplicon 4 (Figures 3 and 4). This methylation expansion was seen only in tumor samples and occurred in both epithelial and stromal cells. In adenoma samples the 5′end of CGI3 remained unmethylated.

The 3′ end of CGI3 (amplicon 6), and the region covered by the neighboring amplicon 7 (located outside CGI3), displayed elevated methylation in normal tissue samples with epithelial cells showing higher methylation levels than stromal cells (Figure 3). In amplicon 7, all epithelial samples were fully methylated while stromal samples were partially methylated. The tumor-associated increase in methylation, which was seen with amplicon 5, was still detected with amplicon 6 but did not affect the region covered by amplicon 7. This pattern indicates the existence of a DNA methylation boundary neighboring CGI3.

In addition to these major methylation patterns in SEPT9, our results show patient-specific differences in the level of methylation (Figure 3). These differences are clearly seen in CGI3 but also seem to occur in CGI2 (amplicon 2). Although this study was based on a small number of patients, the averaging of sequencing data from several individual microdissected specimens (Additional file 2: Table S2) provided a higher degree of robustness against technical noise. Therefore, the subtle differences seen between patients may reflect biological differences especially when they appear in parallel in multiple amplicons (e.g., amplicons 5 and 6). In most stromal cells, amplicon 2 displayed higher methylation levels (up to 25%) in comparison to epithelial cells in all five tissue groups (Figure 3). Amplicon 2 is located within CGI2, which is associated with the transcription start site (TSS) of SEPT9_v1, the longest and possibly the most prominent of the SEPT9 transcripts.

### Immunohistochemistry

**Figure 5:**
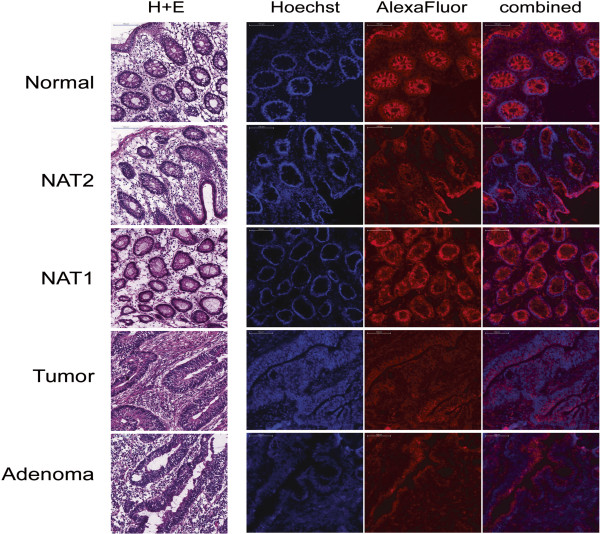
**Histopathology and Septin-9 immunohistochemistry.** The images for H&E staining and IHC were obtained from adjacent sections and are from the same blocks used for LCM sampling. H&E staining in the left panel shows overall tissue morphology. The three IHC images per patient are from one slide: Hoechst staining (blue) marks nuclei, AlexaFluor 546 (red) shows Septin-9 protein; the right panel shows the combination of Hoechst and AlexaFluor 546. For better visualization of the Hoechst staining, these images were obtained with intensified brightness for the blue channel. Normal sections are from BSM0451; NAT1, NAT2, and tumor are from BSM0456, and adenoma is from BSM0457. All images are at 20x magnifications; the scale bars correspond to 100 μm.

**Figure 6:**
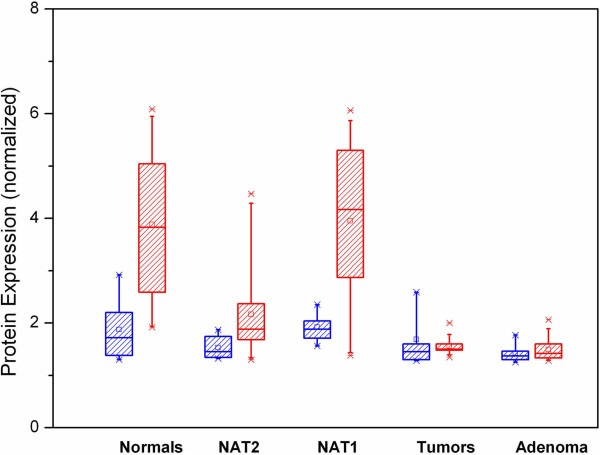
**Quantification of immunohistochemistry results.** Expression of Septin-9 protein in stromal (blue boxes) and epithelial (red boxes) cells. The Y axis displays arbitrary units for the normalized fluorescence signals (ratio AlexaFluor 546 to Hoechst) in IHC slides. Each box plot contains multiple scanned slide sections from three patients.

Antibody staining for Septin-9 was positive in all tissue samples irrespective of disease status (Figure 5). In addition, the protein was found in both epithelial and stromal cells, although, at different levels. Septin-9 protein level was significantly higher in epithelial cells than in stromal cells derived from normal and NAT1 tissues (p <0.001) (Figure 6). In adenoma and tumor samples, the epithelial Septin-9 level was not different from that found in stromal cells (Figure 6). In all five tissue groups, the level of the Septin-9 protein in stromal cells was comparable with the lowest Septin-9 level found in tumor samples.

## Discussion

### Methylation in SEPT9 CGI3

We investigated cell-type specific DNA methylation in eight different regions of the SEPT9 gene in normal, adenoma, and tumor tissues using LCM-separated populations of epithelial and stromal cells. Our results showed major changes in the methylation pattern in diseased tissues in only one of the CpG islands investigated (CGI3). The observed aberrant methylation in adenoma and tumor samples clearly originated in epithelial cells while in stromal cells, hypermethylation at this locus occurred only after hypermethylation in epithelial cells (Figure 3). The results also indicate spreading of aberrant methylation from the core of CGI3 to the 5′ end of the island. This methylation expansion was observed only in CRC samples while in adenoma samples, the 5′end of CGI3 remained clear of methylation indicating that it is a relatively late event in CRC formation. Such a distinctive methylation pattern in the adenoma samples could be described as a transitory methylation status which fits between the normal and tumor samples. Together, these findings indicate that methylation changes in the core of CGI3 of the SEPT9 gene reflect the cellular progression towards malignancy in colon mucosa. These findings imply that alterations in this differentially methylated region (DMR) within SEPT9 represent early events in colon carcinogenesis and may directly contribute to colorectal tumor etiology. Although stromal cells seemed to undergo molecular alterations after changes to epithelial cells, the cells comprising the stromal microenvironment are known to also play an important role in the progression of cancer [30]. Epigenetic differences between epithelial and stromal cells in colon cancer have already been explored in several studies [31-33]. The exact mechanism by which the epigenetic status of one cell is transferred to a neighboring cell is not understood, but it has been hypothesized that multiple elements are involved, such as defects in the epithelial basement membranes, composition of the extracellular matrix, soluble agents (e.g., growth factors and cytokines), and direct cell-to-cell contacts [34,35]. For a neoplastic cell to become invasive, it must first acquire the potential for proteolytic degradation of the basement membrane and actively migrate into the surrounding mesenchymal compartment, which requires the dissolution of intracellular and cell-matrix contacts [34]. Because Septin-9 is involved in cytokinesis, it is possible that a deregulated SEPT9 gene could impact the propensity of a cell for migration and invasiveness. Additionally, accumulating evidence describing the involvement of SEPT9 in the mechanism of epithelial-mesenchymal transition [36] provides further support for such a connection. Considering that transcription of SEPT9 produces several different isoforms, a particular ratio of these transcripts may be needed to sustain normal cellular physiology. If disease-related hypermethylation, such as what we observed with CGI3, suppresses the normal expression of one of these transcripts, differently structured filaments may form affecting key cellular functions [8]. Both the genomic location of CGI3 and the presence of a TSS in this specific region (Figure 1) strongly suggest a direct role in the regulation of transcript variant 2 (tv-2). Experiments are currently underway to determine whether the expression of this or any of the other transcripts is altered in epithelial cells in adenoma and in colorectal cancer. It is of further interest to note that CGI3 also harbors the specific CpGs interrogated by the early CRC detection test, Epi *pro*Colon®, which is currently available for clinical application in Europe [21]. Our findings provide further evidence that Epi *pro*Colon® targets SEPT9 at the most informative site of this biomarker gene.

### Immunohistochemistry

Our results from the IHC analysis of the different tissues indicated a significant reduction of epithelial Septin-9 protein levels in adenoma and tumor tissue. It is tempting to speculate that the increase in SEPT9 methylation of CGI3 in adenoma and tumor samples is directly responsible for the reduced Septin-9 expression levels. However, our current methylation data alone cannot explain the different levels of Septin-9 protein in epithelial and stromal cells, as stromal cells display low levels of Septin-9 protein and their SEPT9 gene is not methylated. Additionally, the increased SEPT9 methylation seen in NAT1 samples and in stromal cells of tumor tissue does not seem to be reflected in a corresponding reduction in protein levels in the same samples. Nevertheless, it is interesting that a reduction of Septin-9 protein levels in epithelial cells was also found in an earlier exploratory study in which a different antibody for Septin-9 detection was used [27]. In our present study, the polyclonal antibody targeted a sequence of 29 amino acids close to the C-terminal end, which is preserved in at least seven Septin-9 isoforms. The antibody used in the previous study targeted a region with a variable length close to the N-terminus detecting only three Septin-9 isoforms. Despite these differences, both studies provided evidence for a reduced Septin-9 protein level in colon adenoma and in CRC. Each of the Septin-9 proteins will need to be targeted simultaneously with highly specific monoclonal antibodies to determine whether the observed aberrant methylation in one CpG island leads to a reduction in protein expression, and if all isoforms are affected equally. Furthermore, histone modifications within SEPT9 may need to be considered in different cell types as these modifications are known to impact the regulation of transcription [37].

### Methylation in additional SEPT9 regions

While CGI3 is a DMR in cells of the colon mucosa, the same cannot be said for other regions analyzed in this study. Earlier studies reported evidence for epigenetic regulation of SEPT9 in ovarian cancer [38] and in head and neck cancer [39], but did not indicate the exact regions involved. Epigenetic regulation of SEPT9 was recently implicated in breast cancer by Connolly et al. [16] who reported increased total SEPT9 mRNA expression and an overall increase in Septin-9 protein levels in breast cancer tissue when compared with normal tissue. In addition, the group found the silencing of a specific transcript variant 3 (tv-3) and the decrease of the Septin-9 protein encoded by tv-3. Elevated methylation in tumor and NAT tissue samples was proposed to trigger these effects. However, the reported region is CpG poor when mapped to the current hg19 build of the human genome, upstream of the first exon for tv-3. The same region is located about 3 kb upstream of amplicon 3 in our study (Figure 1). The reported absolute methylation levels between normal and tumor tissues differed by less than 12%. In comparison, differential methylation in the core of CGI3 seen in our study exceeded 80% in epithelial cells. It remains to be demonstrated whether the same CpG islands in SEPT9 are aberrantly methylated in different tumors and how those CpG islands may impact the expression of the various mRNAs.

### Methylation boundary

The consistently methylated region covered by amplicon 7, positioned approximately 2 kb downstream of CGI3, stood out as a characteristic feature in the SEPT9 methylation landscape of our samples (Figure 3). We compared our results with publicly available next-generation sequencing data [40]. This database also indicated complete methylation in this particular region in all cell types for which data were available. In addition, the presence of a CTCF binding site approximately 100 bp downstream of amplicon 6 further underscores the functional relevance of this region [41]. Supported by these observations, we concluded that the region around amplicon 7 represents a DNA methylation boundary. Stromal cells in this region consistently displayed lower levels of methylation, which further supports our observation of epigenetic differences between stromal and epithelial cell types.

Boundary elements are frequent features of CpG islands, and they define functionally important domains, such as insulators that may block enhancer activities [42]. The boundary elements, however, are not unchangeable features of a particular region. Loss of methylation boundaries have been described in fragile-X syndrome [43], and the shift of boundary elements, either inward or outward of the respective CGI, were reported in colon cancer [44]. Either of these situations leads to a loss of methylation stability in the DMR. Interestingly, while our data indicate the presence of a methylation boundary downstream of CGI3, it seems that the position is not shifted in tumor tissue and does not disappear as the location of this element in the region of amplicons 6 and 7 persists in both adenoma and tumor tissues. Genome-wide methylation analyses of the colon have suggested that local detection of CGI hypermethylation usually reflects only the shift of a boundary element [44]. Additional studies will be required to determine whether this model also applies to SEPT9 or if this CGI is unusual, e.g., it is flanked by a position invariable methylation boundary.

## Conclusions

Our results support the idea that epigenetic deregulation of SEPT9 plays a role in the development of colorectal cancer. Aberrant hypermethylation of this gene occurs only in one of its CpG islands and this hypermethylation likely is an early event in the adenoma-carcinoma sequence. Tumor-associated aberrant methylation in the colon mucosa originates in epithelial cells and precedes similar alterations in stromal cells. Our data further provide a direct link between the region interrogated by the Epi *pro*Colon® assay, which detects methylation of SEPT9 in cell-free DNA in plasma and the underlying events in the tissue of cancer patients. It is now evident that the Epi *pro*Colon® test, currently available for clinical application in Europe, obtains its diagnostic utility by targeting the very region of the SEPT9 promoter that displays the highest susceptibility to methylation changes in the adenoma-carcinoma sequence. Additional applications of this biomarker in oncology, beyond the known utility for diagnostic screening, are also conceivable.

### Endnotes

^a^Throughout this manuscript the human gene septin 9 is described with the gene symbol name “SEPT9” while “Septin-9” is used for the protein, following the recommended nomenclature by Expasy Gene/Protein Synonyms databank and UniProt database.

## Abbreviations

CGI: CpG island; CIMP: CpG island methylator phenotype; CpG: Cytosine guanine dinucleotide; CRC: Colorectal cancer; CTCF: CCCTC-binding factor; DMR: Differentially methylated region; GTP: Guanosine triphosphate; H&E: Hematoxylin and Eosin (stain); IHC: Immunohistochemistry; LCM: Laser capture microdissection; MLL: Myeloid/lymphoid or mixed-lineage leukemia gene; mPCR: Multiplex PCR; NAT: Normal adjacent tissue; OCT: Optimal cutting temperature media; PBL: Peripheral blood lymphocytes; PBS: Phosphate buffered saline; PMR: Percent of methylated reference; qPCR: Quantitative PCR; SEPT9: Septin 9 gene; sPCR: Singleplex PCR; TSS: Transcription start site; tv: Transcription variant.

## Competing interests

RW and MK were employees at Epigenomics AG at the time the study was designed and the experiments were performed. AZS was an employee at and currently is consultant to Epigenomics Inc. RW and AZS are shareholders of Epigenomics. All other authors declare that they have no competing interests.

## Authors’ contributions

BM, MK, and RW conceived the study; MK designed primers and performed a pilot study to establish the approach; SS collected the samples and performed cryosectioning; AK and KT performed LCM and IHC experiments; GV measured and analyzed IHC data; RW supervised and analyzed all methylation related experiments and wrote the manuscript; AZS and ZT contributed to the design and critical review of the manuscript. All authors read and approved the final manuscript.

## Pre-publication history

The pre-publication history for this paper can be accessed here:

http://www.biomedcentral.com/1471-2407/13/398/prepub

## Supplementary Material

Additional file 1: Table S1 Patient characteristics and clinical data. Click here for file

Additional file 2: Table S2 Collected LCM specimens. Click here for file

Additional file 3: Table S3 Primers used for mPCR and sPCR assays. Reverse primers used for locus specific resequencing after mPCR were as shown in the table but had an added ‘cgtcgtcg’-tag at their 5′ end. Click here for file

Additional file 4: Figure S1 Amplicon information. Distribution of CpGs within each of the eight amplicons for SEPT9. All amplicons are drawn to the same scale; the real length of each amplicon, given in base pairs, is shown in the second column, and the number of CpGs covered by the respective amplicon in the third column. Click here for file
