# Supplementary material for: Aberrant septin 9 DNA methylation in colorectal cancer is restricted to a single CpG island
Source: BMC Cancer. 2013 Aug 30;13:398. doi: 10.1186/1471-2407-13-398 (PMC3837632; doi:10.1186/1471-2407-13-398)
Supplement: Additional file 1: Table S1 — Patient characteristics and clinical data. [file 1471-2407-13-398-S1.pdf]

Subject_ID	Diagnosis	Gender	Age	Location	Stage	Grading	PolypSize	Type
BSM 0451	Healthy	F	32	NA	NA	NA	NA	NA
BSM 0452	Healthy	F	72	NA	NA	NA	NA	NA
BSM 0453	Healthy	M	67	NA	NA	NA	NA	NA
BSM 0454	Tumor	M	71	sigmoid	pT2 pN0	G2	NA	adenocarcinoma
BSM 0455	Tumor	M	72	rectosigmoid	pT2 pN1	G2	NA	adenocarcinoma
BSM 0456	Tumor	F	53	cecum	pT3 pN0	G2	NA	adenocarcinoma
BSM 0457	Adenoma	F	75	sigmoid	pT2 pN0	G2	12 mm	tubulovillous
BSM 0458	Adenoma	F	62	sigmoid	pT3 pN1	G3	7 mm	tubular
BSM 0459	Adenoma	M	71	sigmoid	pT2 pN0	G2	15 mm	tubulovillous
