# Supplementary material for: Aberrant septin 9 DNA methylation in colorectal cancer is restricted to a single CpG island
Source: BMC Cancer. 2013 Aug 30;13:398. doi: 10.1186/1471-2407-13-398 (PMC3837632; doi:10.1186/1471-2407-13-398)
Supplement: Additional file 2: Table S2 — Collected LCM specimens. [file 1471-2407-13-398-S2.pdf]

LCM spec. ID	Subject group	patient #	Subject code	cell type	bisDNA yield (ng)	Group description	# of samples averaged in data analysis
1	**Healthy**	patient 1	BSM 0451	Epithelial	9,82	Healthy_patient1_epith	6
2	**Healthy**	patient 1	BSM 0451	Epithelial	8,45		
3	**Healthy**	patient 1	BSM 0451	Epithelial	2,37		
4	**Healthy**	patient 1	BSM 0451	Epithelial	4,32		
5	**Healthy**	patient 1	BSM 0451	Epithelial	3,25		
6	**Healthy**	patient 1	BSM 0451	Epithelial	5,92		
9	**Healthy**	patient 2	BSM 0452	Epithelial	3,49	Healthy_patient2_epith	6
10	**Healthy**	patient 2	BSM 0452	Epithelial	4,42		
11	**Healthy**	patient 2	BSM 0452	Epithelial	10,69		
12	**Healthy**	patient 2	BSM 0452	Epithelial	4,03		
13	**Healthy**	patient 2	BSM 0452	Epithelial	2,24		
14	**Healthy**	patient 2	BSM 0452	Epithelial	4,38		
17	**Healthy**	patient 3	BSM 0453	Epithelial	4,24	Healthy_patient3_epith	6
18	**Healthy**	patient 3	BSM 0453	Epithelial	5,82		
19	**Healthy**	patient 3	BSM 0453	Epithelial	2,98		
20	**Healthy**	patient 3	BSM 0453	Epithelial	1,20		
21	**Healthy**	patient 3	BSM 0453	Epithelial	4,00		
22	**Healthy**	patient 3	BSM 0453	Epithelial	3,17		
7	**Healthy**	patient 1	BSM 0451	Stromal	2,30	Healthy_patient1_strom	2
8	**Healthy**	patient 1	BSM 0451	Stromal	10,23		
15	**Healthy**	patient 2	BSM 0452	Stromal	8,46	Healthy_patient2_strom	2
16	**Healthy**	patient 2	BSM 0452	Stromal	2,11		
23	**Healthy**	patient 3	BSM 0453	Stromal	4,06	Healthy_patient3_strom	2
24	**Healthy**	patient 3	BSM 0453	Stromal	1,89		
25	**Tumor**	patient 4	BSM 0454	Epithelial	28,58	Tumor_patient1_epith	6
26	**Tumor**	patient 4	BSM 0454	Epithelial	18,75		
27	**Tumor**	patient 4	BSM 0454	Epithelial	4,22		
28	**Tumor**	patient 4	BSM 0454	Epithelial	18,83		
29	**Tumor**	patient 4	BSM 0454	Epithelial	9,03		
30	**Tumor**	patient 4	BSM 0454	Epithelial	1,38		
41	**Tumor**	patient 5	BSM 0455	Epithelial	15,58	Tumor_patient2_epith	6
42	**Tumor**	patient 5	BSM 0455	Epithelial	4,93		
43	**Tumor**	patient 5	BSM 0455	Epithelial	6,08		
44	**Tumor**	patient 5	BSM 0455	Epithelial	5,25		
45	**Tumor**	patient 5	BSM 0455	Epithelial	7,30		
46	**Tumor**	patient 5	BSM 0455	Epithelial	17,25		
57	**Tumor**	patient 6	BSM 0456	Epithelial	1,06	Tumor_patient3_epith	6
58	**Tumor**	patient 6	BSM 0456	Epithelial	0,61		
59	**Tumor**	patient 6	BSM 0456	Epithelial	1,28		
60	**Tumor**	patient 6	BSM 0456	Epithelial	1,47		
61	**Tumor**	patient 6	BSM 0456	Epithelial	7,17		
62	**Tumor**	patient 6	BSM 0456	Epithelial	12,58		
31	**Tumor**	patient 4	BSM 0454	Stromal	23,01	Tumor_patient1_strom	2
32	**Tumor**	patient 4	BSM 0454	Stromal	25,22		
47	**Tumor**	patient 5	BSM 0455	Stromal	11,08	Tumor_patient2_strom	2
48	**Tumor**	patient 5	BSM 0455	Stromal	2,50		
63	**Tumor**	patient 6	BSM 0456	Stromal	15,81	Tumor_patient3_strom	2
64	**Tumor**	patient 6	BSM 0456	Stromal	4,35		
33	**NAT1**	patient 4	BSM 0454	Epithelial	1,96	NAT_patient1_distance1_epith	3
34	**NAT1**	patient 4	BSM 0454	Epithelial	10,82		
35	**NAT1**	patient 4	BSM 0454	Epithelial	22,85		
49	**NAT1**	patient 5	BSM 0455	Epithelial	2,34	NAT_patient2_distance1_epith	3
50	**NAT1**	patient 5	BSM 0455	Epithelial	7,89		
51	**NAT1**	patient 5	BSM 0455	Epithelial	1,46		
65	**NAT1**	patient 6	BSM 0456	Epithelial	6,11	NAT_patient3_distance1_epith	3
66	**NAT1**	patient 6	BSM 0456	Epithelial	1,34		
67	**NAT1**	patient 6	BSM 0456	Epithelial	2,08		
36	**NAT1**	patient 4	BSM 0454	Stromal	8,00	NAT_patient1_distance1_strom	1
52	**NAT1**	patient 5	BSM 0455	Stromal	6,21	NAT_patient2_distance1_strom	1
68	**NAT1**	patient 6	BSM 0456	Stromal	1,72	NAT_patient3_distance1_strom	1

**37**	**NAT2**	patient 4	BSM 0454	Epithelial	9,82	NAT_patient1_distance2_epith	3
**38**	**NAT2**	patient 4	BSM 0454	Epithelial	2,86		
**39**	**NAT2**	patient 4	BSM 0454	Epithelial	2,30		
**53**	**NAT2**	patient 5	BSM 0455	Epithelial	3,97	NAT_patient2_distance2_epith	3
**54**	**NAT2**	patient 5	BSM 0455	Epithelial	25,15		
**55**	**NAT2**	patient 5	BSM 0455	Epithelial	0,94		
**69**	**NAT2**	patient 6	BSM 0456	Epithelial	7,49	NAT_patient3_distance2_epith	3
**70**	**NAT2**	patient 6	BSM 0456	Epithelial	0,10		
**71**	**NAT2**	patient 6	BSM 0456	Epithelial	14,75		
**40**	**NAT2**	patient 4	BSM 0454	Stromal	5,90	NAT_patient1_distance2_strom	1
**56**	**NAT2**	patient 5	BSM 0455	Stromal	11,36	NAT_patient2_distance2_strom	1
**72**	**NAT2**	patient 6	BSM 0456	Stromal	2,47	NAT_patient3_distance2_strom	1
**73**	**Adenoma**	patient 7	BSM 0457	Epithelial	24,29	Polyp_patient1_epith	6
**74**	**Adenoma**	patient 7	BSM 0457	Epithelial	4,19		
**75**	**Adenoma**	patient 7	BSM 0457	Epithelial	6,34		
**76**	**Adenoma**	patient 7	BSM 0457	Epithelial	16,40		
**77**	**Adenoma**	patient 7	BSM 0457	Epithelial	6,52		
**78**	**Adenoma**	patient 7	BSM 0457	Epithelial	0,72		
**81**	**Adenoma**	patient 8	BSM 0458	Epithelial	2,21	Polyp_patient2_epith	6
**82**	**Adenoma**	patient 8	BSM 0458	Epithelial	16,10		
**83**	**Adenoma**	patient 8	BSM 0458	Epithelial	3,39		
**84**	**Adenoma**	patient 8	BSM 0458	Epithelial	9,89		
**85**	**Adenoma**	patient 8	BSM 0458	Epithelial	12,86		
**86**	**Adenoma**	patient 8	BSM 0458	Epithelial	4,61		
**89**	**Adenoma**	patient 4	BSM 0454	Epithelial	3,97	Polyp_patient3_epith	6
**90**	**Adenoma**	patient 4	BSM 0454	Epithelial	19,23		
**91**	**Adenoma**	patient 4	BSM 0454	Epithelial	13,98		
**92**	**Adenoma**	patient 4	BSM 0454	Epithelial	2,97		
**93**	**Adenoma**	patient 4	BSM 0454	Epithelial	19,04		
**94**	**Adenoma**	patient 4	BSM 0454	Epithelial	21,54		
**79**	**Adenoma**	patient 7	BSM 0457	Stromal	3,52	Polyp_patient1_strom	2
**80**	**Adenoma**	patient 7	BSM 0457	Stromal	22,48		
**87**	**Adenoma**	patient 8	BSM 0458	Stromal	12,00	Polyp_patient2_strom	2
**88**	**Adenoma**	patient 8	BSM 0458	Stromal	1,61		
**95**	**Adenoma**	patient 4	BSM 0454	Stromal	12,00	Polyp_patient3_strom	2
**96**	**Adenoma**	patient 4	BSM 0454	Stromal	5,62		
