# Supplementary material for: Aberrant septin 9 DNA methylation in colorectal cancer is restricted to a single CpG island
Source: BMC Cancer. 2013 Aug 30;13:398. doi: 10.1186/1471-2407-13-398 (PMC3837632; doi:10.1186/1471-2407-13-398)
Supplement: Additional file 3: Table S3 — Primers used for mPCR and sPCR assays. Reverse primers used for locus specific resequencing after mPCR were as shown in the table but had an added ‘cgtcgtcg’-tag at their 5′ end. [file 1471-2407-13-398-S3.pdf]

Locus	Primer ID	Sequence 5' - 3'	Amplicon mapping
SEPT9_Amp 1	S9_reg1_F	TGTGGAGGGGTTTAGGTGT	chr17:75,242,851..75,243,008
	S9_reg1_R	AATACCCCCTTCTCAACACA	
SEPT9_Amp 2	S9_reg2_F	TGGAGATTTTAAGGATATTTAAGTAGAGA	chr17:75,276,696..75,276,963
	S9_reg2_R	ACACCTATCCTACTACTTTTCTAATAATCA	
SEPT9_Amp 3	S9_reg3_F	GTTGGGGTATAGGGTGAAGAA	chr17:75,315,725..75,315,893
	S9_reg3_R	CAACTCACAAAATCAAATTCCTAA	
SEPT9_Amp 4	S9_reg4_F	TGGTGGGGGTGTTAGTTGT	chr17:75,368,814..75,369,020
	S9_reg4_R	ACAACCAAACAAAATTCTCTATCAC	
SEPT9_Amp 5	S9_reg5_F	TTTATTTAGTTGAGTTAGGGGGTTTA	chr17:75,369,420..75,369,648
	S9_reg5_R	AACCCAACACCCACCTTC	
SEPT9_Amp 6	S9_reg6_F	TATTTAGGTTTGGGGATATTTTTATT	chr17:75,370,258..75,370,549
	S9_reg6_R	AAACCATTATATAAACTTCCCCTTC	
SEPT9_Amp 7	S9_reg7_F	TTTGTTTGTGAAGATTATATGGGTTA	chr17:75,372,308..75,372,507
	S9_reg7_R	AATTTCCTCCCCACCCTT	
SEPT9_Amp 8	S9_reg8_F	GATTATAAAGTTTTTTTGATTTTTTGTT	chr17:75,447,506..75,447,783
	S9_reg8_R	CACCCCTCTCCTAAAAACC	
SFRP1	SFRP1_F	GGGGAATTTGTTATATTTAAGTATTT	chr8:41,166,196..41,166,356
	SFRP1_R	ACACCCAAATCTTCCTCTACTC	
NEUROG1	NEUROG1_F1	TGGAGTAAGTTTTTGGGTAGGTT	chr5:134,870,698..134,870,971
	NEUROG1_R1	CCTACAACTACATCTAAACTCTAA	
RASSF2	RASSF2_F	CAATCTCCCTATAAAACCACTAACA	chr20:4,803,171..4,803,473
	RASSF2_R	GGATTTTTTAGAGGTAGGTTTTAGTTT	
VIM	VIM_F	AAATCCAATCCTCTACCACTCT	chr10:17,271,267..17,271,436
	VIM_R	GGATTTGGTGGATATGGTTG	
LIMK1	LIMK1_F1	GGTTTATAGGGTTTTTTTAGTTTTG	chr7:73,497,710..73,497,919
	LIMK1_R1	CCTTCATTCATTCTTCTTTCTC	
