# Supplementary figures and images for: Aberrant septin 9 DNA methylation in colorectal cancer is restricted to a single CpG island
Source: BMC Cancer. 2013 Aug 30;13:398. doi: 10.1186/1471-2407-13-398 (PMC3837632; doi:10.1186/1471-2407-13-398)

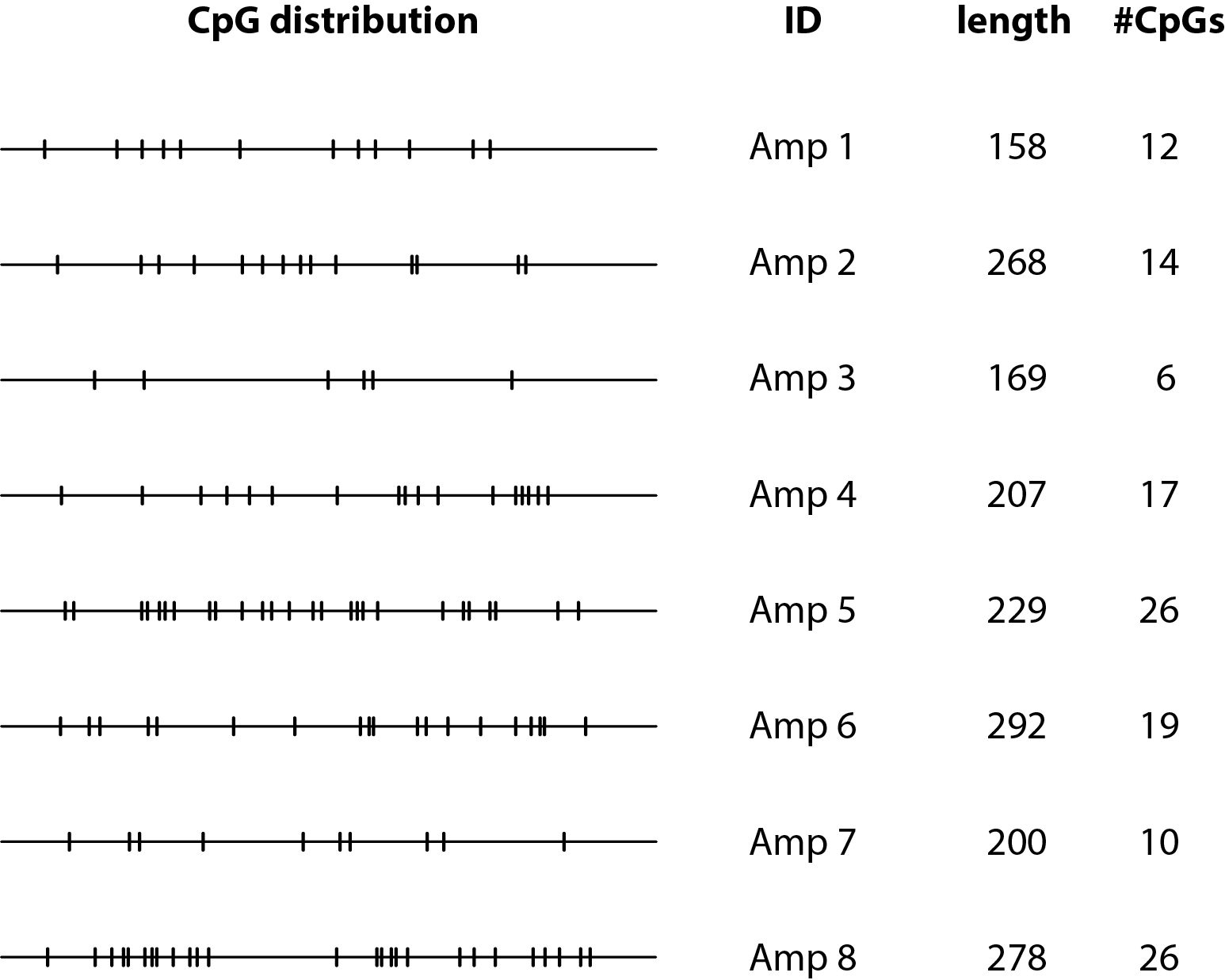

Supplement: Additional file 4: Figure S1 — Amplicon information. Distribution of CpGs within each of the eight amplicons for SEPT9. All amplicons are drawn to the same scale; the real length of each amplicon, given in base pairs, is shown in the second column, and the number of CpGs covered by the respective amplicon in the third column. [file 1471-2407-13-398-S4.jpeg]
